# Supplementary material for: Leucine Zipper Downregulated in Cancer-1 Interacts with Clathrin Adaptors to Control Epidermal Growth Factor Receptor (EGFR) Internalization and Gefitinib Response in EGFR-Mutated Non-Small Cell Lung Cancer
Source: Int J Mol Sci. 2024 Jan 23;25(3):1374. doi: 10.3390/ijms25031374 (PMC10855387; doi:10.3390/ijms25031374)
Supplement: Supplementary file 1 [file ijms-25-01374-s001.zip › ijms-2747289-supplementary.pdf]

Title: LDOC1 interacts with clathrin adaptors to controls EGFR internalization and gefitinib response in EGFR-Mutated Non–Small Cell Lung Cancer

Journal: International Journal of Molecular Science

Authors: Hsien-Neng Huang, Pin-Feng Hung, Yai-Ping Chen, Chia-Huei Lee*

Corresponding author: C.H.L.: chlee124@nhri.org.tw ; National Institute of Cancer Research, National Health Research Institutes, No. 35, Keyan Road, Zhunan Town, Miaoli 35053, Taiwan.

Supplementary Information

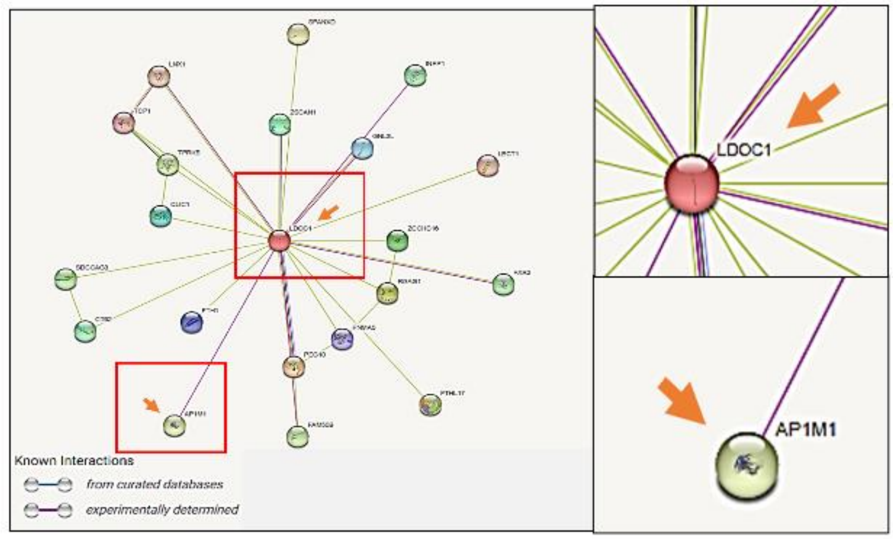

Fig. S1 STRING database predicts interaction between LDOC1 and AP1M1 [STRING: functional protein association networks (string-db.org) version 10.0]. Red arrows indicate LDOC1 and APIM1.

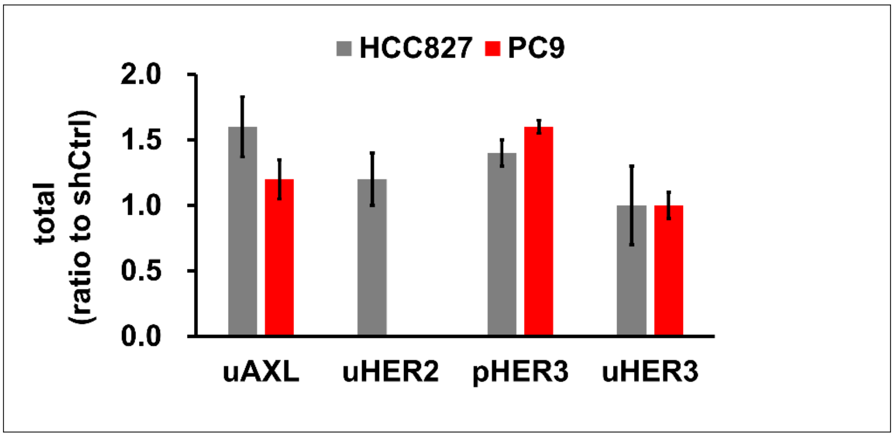

Fig. S2 The summary of the semiquantitative data regarding total protein lysates shown in Figure 3.

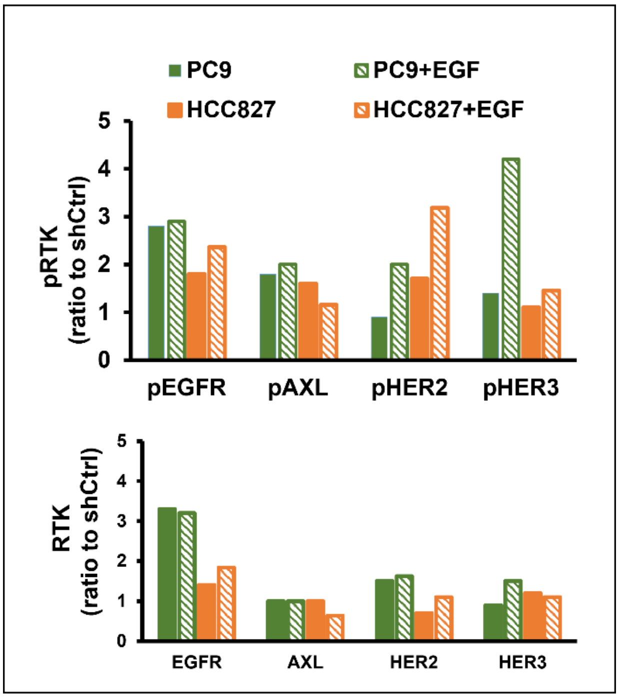

Fig. S3 Semiquantitative evaluation **of the effect of** LDOC1 depletion on the expression of phosphorylated (p) and unphosphorylated (u) EGFR in PC9 and HCC827 cells.
